# Metabolic Differences in 24-Hour Urine Parameters Between Calcium Oxalate Monohydrate and Dihydrate Kidney Stones: A Clinical Study

**DOI:** 10.3390/diagnostics15080994

**Published:** 2025-04-14

**Authors:** Nariman Gadzhiev, Vitaliy Gelig, Gennadii Rodionov, Vineet Gauhar, Guohua Zeng

**Affiliations:** 1 Department of Urology, Saint-Petersburg State University Hospital, 190103 Saint Petersburg, Russia; vgelig@yandex.ru; 2 Center for Transgenesis and Genome Editing, Saint-Petersburg State University, 199034 Saint Petersburg, Russia; 3 The Nikiforov Center of Emergency and Radiation Medicine, 197082 Saint Petersburg, Russia; 4 Division of Urology, Department of General Surgery, Ng Teng Fong General Hospital, National University Health System, Singapore 609606, Singapore; 5 Department of Urology and Guangdong Key Laboratory of Urology, The First Affiliated Hospital of Guangzhou Medical University, Guangzhou 510230, China

**Keywords:** kidney stone disease, calcium oxalate, whewellite, weddellite, 24 h urine specimen

## Abstract

**Background:** Different types of kidney stones are associated with distinct changes in urine chemistry. **Methods:** We assessed urinary parameters of 98 patients with calcium oxalate (CaOx) stones one month following endoscopic stone removal. The 24 h urine analysis encompassed the assessment of various parameters, including volume, sodium, chloride, sulfate, nitrate, fluoride, phosphate, calcium, potassium, magnesium, oxalate, uric acid, citrate, creatinine, and pH levels. **Results:** Hypocitraturia was the most prevalent urinary abnormality (61.2%, *n* = 63), followed by low urine volume (53%, *n* = 52) and hypercalciuria (50%, *n* = 49). We did not find any statistically significant differences between patients with whewellite (COM) (*n* = 69) and weddellite (COD) stones (*n* = 29) (*p* > 0.05). However, oxalate concentration was the only parameter with a statistically significant intergroup difference (*p* = 0.0297). Additionally, in univariate linear regression analysis, urinary phosphate levels ≥ 48.0 mmol/d showed a trend towards significance (OR 0.17, 95% CI 0.02–1.15, *p* = 0.0692), indicating that phosphaturia is associated with a significant increase in the odds ratio of COD stones. To further explore metabolic heterogeneity among stone formers, we conducted cluster analysis, which revealed three distinct metabolic subgroups. Cluster 1 was predominantly associated with COM stones (80.5%) and exhibited significantly higher urinary excretion of sodium, calcium, oxalate, phosphate, and uric acid compared to Cluster 2, which had a more balanced distribution of monohydrate and dihydrate stones. **Conclusions:** These findings suggest that a specific metabolic phenotype may be linked to COM stone formation, providing a framework for risk stratification and personalized prevention strategies in calcium oxalate stone formers.

## 1. Introduction

Kidney stone disease is a prevalent condition affecting a significant portion of the global population and posing significant public health challenges [1]. The lifetime risk of developing kidney stones is estimated to be around 10–15%, with a recurrence rate of approximately 50% within 5 years and 80–90% within 10 years [2,3]. Surgical treatment of urolithiasis in most cases does not prevent disease recurrence, which can impair kidney function, increase treatment costs, reduce quality of life, and may require patients to take time off work, thereby further elevating both public and private healthcare expenses [4]. This highlights the need for effective prophylactic management strategies, based on metabolic evaluation, including stone analysis, blood tests and 24 h urine collection, to mitigate the risk of recurrence [5].

The European Association of Urology (EAU) recommends stone analysis for all first-time stone formers and repeated stone analysis in the case of recurrence under pharmacological prevention or early recurrence after interventional therapy with complete stone clearance [6]. Interestingly, stone composition can also inform the scope of metabolic evaluation. For instance, patients with non-calcium-based stones, such as uric acid or struvite stones, often exhibit distinct metabolic profiles, suggesting that extensive screening, including 24 h urine collection, may not always be necessary [7]. On the other hand, for calcium oxalate (CaOx) stones, which account for nearly 80% of all kidney stones, metabolic evaluation plays a pivotal role in recurrence prevention. To optimize the accuracy of 24 h urine analysis, guidelines recommend that patients should ideally be stone-free for at least twenty days prior to sample collection, since this period minimizes the influence of surgical interventions or acute episodes on metabolic profiles [8].

The morpho-constitutional classification system proposed by M. Daudon et al. has further advanced our understanding of kidney stones by categorizing them into 7 main types and 22 subtypes, each associated with specific urinary chemistry patterns [9,10]. Recognizing these differences is essential for designing effective preventive strategies. Although the exact pathophysiology of stone formation remains unclear, the critical initial step is the supersaturation of urine with stone-forming constituents [11]. For instance, CaOx monohydrate stones (COM), also known as whewellite, classified under stone type 1, are primarily caused by hyperoxaluria in the absence of concurrent hypercalciuria. Conversely, CaOx dihydrate stones (COD), also known as weddellite, fall under stone type 2, and predominantly linked to hypercalciuria, occurring in approximately 85% of cases [12].

While these distinctions in urinary chemistry are well documented, they are not always readily apparent in routine clinical practice, raising questions about their validity and clinical relevance. CaOx stones, accounting for nearly 80% of all kidney stones, are of particular interest due to their high prevalence and significant clinical impact. We questioned whether measurable differences in 24 h urine composition exist between patients with COM stones and those with COD stones in the post-surgical period. By identifying potential metabolic distinctions, we hope to contribute to the optimization of stone prevention strategies, ultimately reducing recurrence rates and improving patient care.

## 2. Materials and Methods

From 2023 to 2024, after Institutional Review Board approval, 24 h urine samples were collected from 98 patients with CaOx stones one month following endoscopic stone removal. Patients were distributed into two groups: Group A consisted of 69 patients with COM, and Group B included 29 patients with COD. The stone types were determined by infrared spectroscopy based on a composition predominance exceeding 50%. To determine the 24 h urine composition, an Agilent 7100 capillary electrophoresis system (Agilent Technologies, Clara, CA, USA) with a diode array detector with a wavelength range of 190–600 nm was used.

Urine samples were collected on a random diet following strict protocols to ensure consistency. Patients received detailed instructions to collect all urine output over a 24 h period, using collection kits designed to minimize contamination and bacterial growth. These kits included containers maintained at low temperatures (below 8 °C) to preserve the integrity of the sample and to minimize bacterial growth. Urine pH was measured immediately upon collection using dipsticks.

The 24 h urine analysis encompassed the assessment of various parameters, including volume, sodium, chloride, sulfate, nitrate, fluoride, phosphate, calcium, potassium, magnesium, oxalate, uric acid, citrate, creatinine, and pH levels. Participants received a urine collection kit, which included collecting bottles and pH measuring dipsticks.

***Main specific urinary conditions were defined as follows:***

Hypercalciuria: Urinary calcium excretion exceeding 6.25 mmol/24 h for men and 5 mmol/24 h for women.

Hyperuricosuria: Urinary uric acid excretion above 4.8 mmol/24 h for men and 4.2 mmol/24 h for women.

Hypocitraturia: Urinary citrate excretion below 2.4 mmol/24 h for men and 2.9 mmol/24 h for women.

Hyperoxaluria: Urinary oxalate excretion greater than 0.45 mmol/24 h for both genders.

Exclusion criteria included patients under 18 years of age and those enrolled in a specific stone recurrence prevention program, and patients with incomplete 24 h urine collections or insufficient sample volumes were also excluded to ensure the accuracy of the analysis.

### Statistical Analysis

Quantitative variables were assessed for normality using the Kolmogorov–Smirnov test with Lilliefors correction. Quantitative data are presented as medians and 25th and 75th percentiles. The Mann–Whitney U test was used to compare two groups based on quantitative characteristics. Fisher’s exact test was used to compare categorical variables, as it is more appropriate for small sample sizes and provides accurate *p*-values when expected frequencies in any cell are less than five. Binary logistic regression was used to assess the relationship between parameters and stone composition. The variables with a significance of *p*-level <0.1 in the univariate model were included in the multivariate analysis with adjusted parameters. For each variable, the odds ratio (OR) and 95% confidence interval (95% CI) were calculated. Statistical significance was determined at a *p*-level <0.05. A K-Means cluster analysis was performed to group the observations into homogeneous clusters. The optimal number of clusters was determined using the Cubic Clustering Criterion (CCC). Prior to clustering, all quantitative variables were standardized. Clustering was performed in the space of principal components, with the first and third principal components (PC1 and PC3) used for visualization. The clustering results were validated by analyzing differences in biochemical parameters between clusters using the Kruskal–Wallis test and Mann–Whitney U test. Analysis of data was performed using the GraphPad Prism v.10 Software.

## 3. Results

A total of 98 patients had available 24 h urine collections and were included in the analysis. Hypocitraturia was the most common urinary abnormality, 61.2 % (*n* = 63), followed by low urine volume (*n* = 52, 53%), hypercalciuria (*n* = 49, 50%), hyperuricosuria (*n* = 29, 29.5%), and hyperoxaluria (*n* = 21, 21.4%).

After dividing the data into groups, we compared urinary parameters and found no statistically significant differences (*p* > 0.05), except for age (Table 1). However, this difference was deemed clinically insignificant.

**Figure 1:**
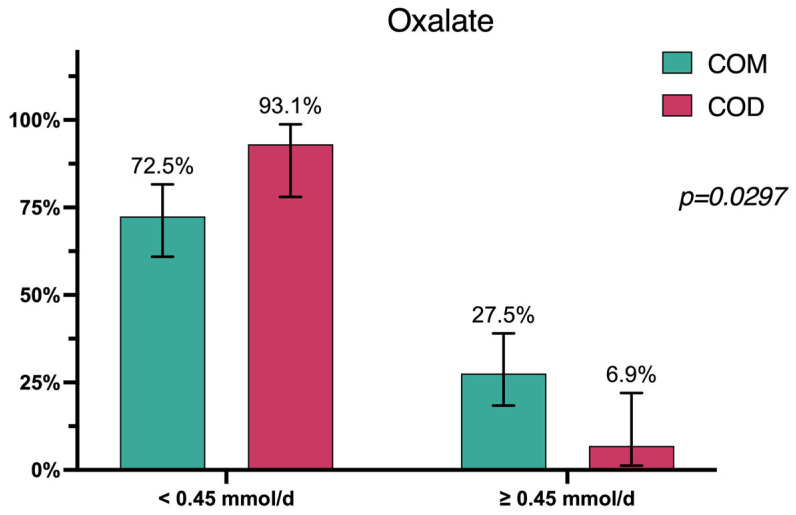
Patient distribution in accordance with urine oxalate concentration levels.

Assessing the proportion of patients whose parameter values deviated from the designated reference values between the groups, we found that oxalate concentration was the only parameter that had statistically significant intergroup differences (*p* = 0.0297) with 27.5% of patients (*n* = 19, 95% CI 18.4–39.1) having hyperoxaluria in Group A vs. 6.9% (*n* = 2, 95% CI 1.2–22.0) in Group B (Figure 1).

We performed univariable regression analysis of the dataset. The parameters that showed a significant association at the *p* < 0.1 level were identified—age, diuresis, ammonium, sodium, oxalate, pH, and phosphate (Table 2).

**Figure 2:**
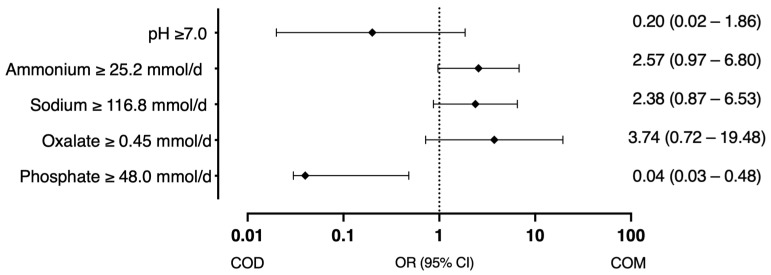
Factors associated with the probability of COD and COM stones (odds ratio with 95% CI).

Phosphate ≥ 48.0 mmol/d (encountered in 15.3% of patients) showed a trend towards significance in the univariate model (OR 0.17, 95% CI 0.02–1.15, *p* = 0.0692), and when adjusted for sex, age and diuresis was statistically significant factor in the adjusted model (OR 0.04, 95% CI 0.03–0.48, *p* = 0.012), indicating that phosphaturia is associated with a significant increase in the odds ratio of COD stones (Figure 2).

**Figure 3:**
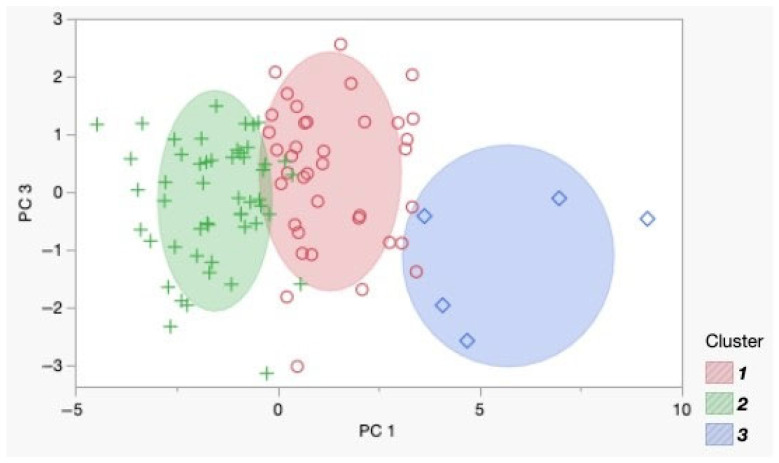
Biplot diagram of the identified clusters in the principal component coordinates. Each point represents an individual observation: green crosses correspond to Cluster 1, red circles to Cluster 2, and blue squares to Cluster 3.

To identify patterns and group observations, a cluster analysis was performed, which revealed three clusters (*N* = 3), determined by the optimal CCC value of 3.7757. The data were pre-normalized for accurate centroid determination. The final distribution of observations was Cluster I—41 objects; Cluster II—51 objects; and Cluster III—6 objects. A Biplot diagram visualized the results (Figure 3).

To further investigate the characteristics of the identified clusters, we analyzed the composition of urinary stones within each cluster (Table 3).

It was found that Cluster 1 represented a phenotypic group primarily forming COM stones (80.5%), whereas Cluster 2 was a mixed group, with a more balanced distribution between monohydrate and dihydrate stones. This suggested a metabolic profile that favored monohydrate stone formation in Cluster 1. To assess whether the clusters differed in metabolic urine parameters, we compared Cluster 1 and Cluster 2. Cluster 3 was excluded from the analysis due to the small number of patients (Table 4).

Cluster 1 exhibited a distinct metabolic profile with significantly higher excretion of key stone-forming substances, including sodium, calcium, oxalate, phosphate, and uric acid, compared to Cluster 2. These findings suggest that Cluster 1 may have a greater predisposition to calcium–oxalate stone formation, particularly of the COM type, which was predominant in this group.

## 4. Discussion

Kidney stone disease is a widespread condition that imposes a significant healthcare burden, affecting approximately 10–15% of the global population, making it the most common urological disorder. Additionally, nearly half of patients with nephrolithiasis will experience a recurrence within a decade [13]. Recurrent stone formers may benefit from metabolic evaluation through 24 h urine stone risk parameter studies. This testing identifies specific metabolic abnormalities, allowing clinicians to tailor pharmacologic and dietary interventions to reduce the likelihood of future stone formation [14,15].

The role of 24 h urine testing in urologic practice is important as both the American Urological Association (AUA) and EAU Guidelines recommend it, especially in high-risk stone formers [16]. However, despite these guideline directives, the overall prevalence of 24 h urine collection use in clinical practice remains remarkably low. For instance, among privately insured Americans identified as high risk for stone recurrence, only 7% undergo this testing. Of those tested, only 16% with an initial abnormality undergo repeat collections within six months. Notably, the likelihood of metabolic evaluation is 2.9 times higher when a patient is seen by a nephrologist and more than threefold higher when seen by a urologist [17,18].

The 24 h urine analysis can often be inferred from stone composition data, particularly with the aid of morpho-constitutional classification developed by Prof. Michel Daudon. This system accurately identifies and structures all lithogenic factors responsible for stone formation and recurrence. According to the classification, all stones are divided into 7 main types and 22 subtypes. COM, classified as Stone Type 1, is primarily associated with hyperoxaluria without concurrent hypercalciuria. In contrast, COD, classified as Stone Type 2, is predominantly linked to hypercalciuria, occurring in approximately 85% of cases [19]. However, these differences in urine composition are not consistently evident in clinical practice among CaOx stone formers [20]. CaOx is the most prevalent chemical component of urinary stones worldwide, making it the point of our interest.

Our study revealed that hypocitraturia was the most prevalent urinary abnormality (61.2%, *n* = 63), followed by low urine volume (53%, *n* = 52) and, last, hypercalciuria (50%, *n* = 49). This observation aligns well with the findings of Wu W. et al. [20], as well as with other previous studies that have identified hypocitraturia and low urine volume as some of the most prevalent urinary abnormalities among CaOx kidney stone formers [21,22]. We compared urinary parameters between patients with COM stones (Group A) and those with COD stones (Group B) and found no statistically significant differences overall (*p* > 0.05). However, oxalate concentration was the only parameter with a statistically significant intergroup difference, with 27.5% of patients in Group A exhibiting hyperoxaluria compared to 6.9% in Group B (*p* = 0.0297).

This finding was consistent with the results of Trinchieri et al. [23], who also did not observe higher urinary oxalate excretion in patients with COM stones compared to those with COD stones. One possible explanation for the discrepancies in the prevalence of urinary abnormalities could be the variation in reference values used by different sources such as the EAU, Laboratory Corporation of America Holdings (LabCorp), or Tietz Textbook of Laboratory Medicine [8,24,25]. Each of them may define normal ranges for urinary parameters differently, which can impact the interpretation of results and the reported prevalence of metabolic abnormalities among patients. By selecting LabCorp’s values, we aimed to maintain consistency in our analysis while recognizing that the choice of reference values could influence the reported frequency of urinary abnormalities and, consequently, the recommended treatment strategies (Table 5).

In univariate linear regression analysis, urinary phosphate levels ≥ 48.0 mmol/d showed a trend towards significance (OR 0.17, 95% CI 0.02–1.15, *p* = 0.0692). When adjusted for sex, age, and diuresis, phosphaturia emerged as a statistically significant factor (OR 0.04, 95% CI 0.03–0.48, *p* = 0.012), indicating a significant association with an increased likelihood of COD stone formation. Among our patient cohort, phosphaturia was detected in 15.3% (*n* = 15). This finding is consistent with the 19.9% incidence of hyperphosphaturia reported by Yun-Sok Ha et al., who also demonstrated that hyperphosphaturia is an independent predictor of recurrent stone formation in first-time stone formers, with a hazard ratio (HR) of 2.1 (95% confidence interval [CI]: 1.100–4.097, *p* = 0.025) [26]. It can be explained by the fact that renal phosphate leakage and the associated phosphaturia result in increased production of 1,25(OH)2-vitamin D3, which enhances intestinal absorption of phosphate and calcium.

The combination of hyperphosphaturia and hypercalciuria due to increased intestinal calcium absorption facilitates the formation of calcium phosphate complexes, leading to urolithiasis [27]. Additionally, early research by Tozuka, K et al. indicated that COD stones have a significantly higher apatite content than COM stones [28]. In clinical practice, these observations are often overlooked. However, we emphasize that for evaluating kidney stone patients, it is essential to conduct a precise analysis of the stone to determine its exact composition and 24 h urine analysis utilizing proper reference values.

Given that not all patients with COM stones exhibit hyperoxaluria, we aimed to determine whether a distinct phenotype exists among patients with CaOx stones [29]. To explore this, we conducted cluster analysis to identify metabolic patterns among observations. Our analysis revealed three clusters, with Cluster 1 primarily forming COM stones (80.5%), while Cluster 2 displayed a more balanced distribution between monohydrate and dihydrate stones.

To determine whether these clusters correspond to distinct metabolic phenotypes, we compared their 24 h urine parameters. We found that Cluster 1 was characterized by significantly higher urinary excretion of sodium, calcium, oxalate, phosphate, and uric acid compared to Cluster 2, suggesting a metabolic profile that favors monohydrate stone formation. The observed difference in 24 h urinary creatinine between Clusters 1 and 2 most likely reflects the differing sex distribution between these groups, with higher values in Cluster 1 corresponding to a greater proportion of male patients. This aligns with established sex-specific reference ranges and supports the interpretation that creatinine output in our cohort is consistent with expected physiological differences rather than representing a metabolic abnormality. These findings suggest that specific metabolic alterations may be associated with different calcium oxalate stone compositions. Identifying such phenotypic groups could provide valuable insights for tailored prevention and treatment strategies in calcium oxalate stone formers.

In addition, our analysis revealed that patients with COD stones were significantly younger than those with COM stones (median age 36 vs. 44 years, *p* < 0.0001). This age disparity may reflect distinct pathophysiological processes influencing stone composition. Younger patients might exhibit higher urinary calcium excretion or other metabolic traits favoring COD formation, as suggested by prior studies [30]. Notably, while the difference is statistically significant, both groups fall within the young adult age range (25–44 years) as defined by the World Health Organization. However, even within this cohort, subtle metabolic variations linked to age could contribute to compositional differences. Further investigation into age-related metabolic profiles and their interaction with stone type is warranted to clarify these associations and their clinical implications.

Our study is not devoid of limitations. First, we acknowledge that the relatively small sample size is a limitation of this study, which may affect the generalizability of our findings. This constraint was largely due to our strict inclusion and exclusion criteria designed to ensure data consistency and eliminate confounding factors. Second, being a single-center study, the findings may be influenced by specific local factors, including dietary habits and healthcare practices, that are not representative of other regions. Third, we did not use supersaturation indexes, as prior research by Omar M. et al. has indicated that 24 h urine supersaturation has limited accuracy in predicting the predominant stone composition [31]. Finally, although adjustments were made for certain variables, other potential confounders such as diet, medication adherence, and genetic predispositions were not fully controlled, which could influence the results.

Despite this, we believe the strength of our analysis lies in the application of unsupervised multivariate clustering, which allowed us to identify distinct metabolic subtypes among CaOx stone formers. These findings offer a novel perspective on risk stratification and may help guide personalized prevention strategies. Nonetheless, to confirm and expand upon these observations, larger, multi-center studies with longitudinal follow-up are warranted.

Identifying meaningful, modifiable risk factors in clinical settings is crucial for reducing the prevalence of recurrent stone formation. Our findings confirm that patients with COM stones tend to have a higher frequency of hyperoxaluria. Similarly, our results demonstrate that hyperphosphaturia is linked to COD stones. In this context, manipulating with dietary oxalate intake or renal phosphate excretion could be beneficial for prevention of CaOx stones. For instance, thiazide administration, which reduces calcium and phosphate excretion, may help prevent recurrent COD stones. Effectively controlling phosphaturia in patients with hyperphosphaturia may restore normal urine composition and reduce the incidence of stone episodes. Future research should focus on larger, multi-center studies to validate these findings and further refine personalized treatment approaches for recurrent stone formers.

## 5. Conclusions

Hyperoxaluria is predominantly seen in patients with COM stones, whereas hyperphosphaturia significantly correlates with the formation of COD stones. These findings highlight the importance of incorporating targeted dietary and pharmacological strategies to address specific metabolic abnormalities for effective prevention and management of kidney stones. Furthermore, our cluster analysis revealed distinct metabolic subgroups among CaOx stone formers, suggesting that patients with COM stones may exhibit a unique metabolic phenotype characterized by higher urinary excretion of sodium, calcium, oxalate, phosphate, and uric acid. Identifying such phenotypic patterns can improve risk stratification and enable more personalized treatment approaches, optimizing stone prevention strategies based on the metabolic profile of each patient.

## Figures and Tables

**Table 1:** Baseline characteristics between the groups, including urinary parameters, between the groups.

Parameters	Me [LQ; UQ] (*N* = 69) COM	Me [LQ; UQ] (*N* = 29) COD	*p*-Value	Reference Range
Age	44.00 [37.00; 56.00]	36.00 [31.00; 47.00]	<0.0001	
Male, *n* (%)	48 (69.6)	22 (75.9)	0.6283	
Female, *n* (%)	21 (30.4)	7 (24.1)		
Diuresis, L	2.00 [1.50; 2.50]	1.60 [1.20; 2.00]	0.0606	>2.0
pH level	6.00 [6.00; 6.50]	6.50 [6.00; 6.50]	0.2619	5.0–7.0
Ammonium	27.48 [19.20; 34.56]	23.13 [16.96; 30.40]	0.0704	15.0–60.0 mmol/d
Sodium	168.15 [116.81; 250.05]	120.96 [92.64; 190.19]	0.1510	50.0–150.0 mmol/d
Potassium	44.60 [35.38; 60.90]	48.18 [36.57; 61.45]	0.9876	20.0–100.0 mmol/d
Magnesium	4.25 [2.80; 6.06]	3.30 [2.70; 4.75]	0.1566	1.25–5.0 mmol/d
Calcium	5.71 [3.80; 8.40]	6.00 [4.54; 9.44]	0.5033	male < 5.0; female < 6.25
Chloride	141.28 [87.88; 182.85]	119.50 [87.62; 195.93]	0.7526	70.0–250.0 mmol/d
Nitrate	0.80 [0.42; 1.43]	0.81 [0.53; 1.62]	0.5489	0.1–3.3 mmol/d
Sulfate	17.83 [15.08; 24.59]	17.55 [14.06; 26.88]	0.6047	14.0–35.0 mmol/d
Fluoride	0.66 [0.32; 1.50]	0.68 [0.44; 1.41]	0.8154	0.2–5.5 mmol/d
Phosphate	25.80 [21.42; 31.75]	25.75 [23.21; 31.64]	0.8824	19.0–39.0 mmol/d
Citrate	2.14 [1.26; 2.98]	1.99 [1.39; 2.85]	0.8886	male > 2.4; female > 2.9
Oxalate	0.28 [0.18; 0.45]	0.28 [0.24; 0.33]	0.4314	<0.45 mmol/d
Uric acid	3.57 [2.18; 4.80]	3.15 [2.59; 4.99]	0.6266	male < 4.8; female > 4.2
Creatinine	13.20 [9.60; 15.40]	14.70 [11.10; 16.40]	0.2813	male13.0–18.0; female 7.0–13.0

**Table 2:** Characteristics of the variables that are associated with COM stones.

Variable	OR * (95% CI)	*p*-Value	OR ** (95% CI)	*p*-Value
Age	1.06 (1.02–1.11)	**0.0070**	-	-
Diuresis	1.95 (0.95–4.01)	0.0695	-	-
Ammonium ≥ 25 mmol/d	2.78 (1.13–6.87)	**0.0265**	2.57 (0.97–6.80)	0.0566
Sodium ≥ 116.8 mmol/d	2.85 (1.15–7.10)	**0.0240**	2.38 (0.87–6.53)	0.0925
Oxalate ≥ 0.45 mmol/d	3.06 (0.82–11.34)	0.0945	3.74 (0.72–19.48)	0.1175
pH ≥ 7.0	0.11 (0.01–0.95)	0.0450	0.20 (0.02–1.86)	0.1583
Phosphate ≥ 48.0 mmol/d	0.17 (0.02–1.15)	0.0692	0.04 (0.03–0.48)	**0.012**

*—unadjusted; **—adjusted (age, sex, and diuresis).

**Table 3:** Distribution of calcium oxalate stone composition across clusters.

Stone Composition	Cluster 1, (*N* = 41)	Cluster 2, (*N* = 51)	Cluster 3, (*N* = 6)	*p*-Level, (df = 2)
**COM**	33 (80.5%)	34 (66.7%)	2 (33.3%)	**0.0428**
**COD**	8 (19.5%)	17 (33.3%)	4 (66.7%)	

**Table 4:** Comparison of metabolic urine parameters between Cluster 1 and Cluster 2.

Variable	Me [LQ; UQ] (*N* = 41) 1	Me [LQ; UQ] (*N* = 51) 2	*p*-Level
PH Urine	6.00 [6.00; 6.50]	6.00 [6.00; 6.50]	0.6294
Ammonium mmol/d	32.64 [25.46; 39.33]	21.85 [14.78; 26.94]	<0.0001
Sodium mmol/d	237.44 [176.12; 278.95]	103.50 [86.77; 128.49]	<0.0001
Potassium mmol/d	53.10 [40.92; 68.08]	41.86 [33.74; 54.15]	0.0063
Magnesium mmol/d	5.04 [3.70; 6.45]	3.00 [2.33; 3.64]	<0.0001
Calcium mmol/d	6.97 [5.08; 9.24]	4.63 [3.38; 6.92]	0.0010
Chloride mmol/d	177.60 [151.68; 219.00]	87.88 [71.68; 103.18]	<0.0001
Nitrate mmol/d	0.91 [0.44; 1.80]	0.72 [0.44; 1.07]	0.1275
Sulfate mmol/d	23.69 [18.20; 27.47]	15.08 [11.29; 17.47]	<0.0001
Fluoride mmol/d	0.68 [0.54; 1.50]	0.56 [0.27; 1.07]	0.1778
Phosphate mmol/d	28.80 [25.47; 34.01]	23.21 [17.62; 28.04]	<0.0001
Citrate mmol/d	1.90 [1.35; 2.80]	2.08 [1.18; 3.07]	0.7474
Oxalate mmol/d	0.40 [0.26; 0.56]	0.25 [0.20; 0.33]	0.0010
Uric acid mmol/d	3.82 [2.51; 5.33]	2.94 [2.18; 3.66]	0.0128
Creatinine mmol/d	15.10 [13.90; 17.95]	11.00 [8.60; 12.60]	<0.0001
Ox/Cr ratio	0.02 [0.02; 0.03]	0.02 [0.02; 0.03]	0.9687
Ph/Cr ratio	1.85 [1.69; 2.40]	2.09 [1.65; 2.69]	0.2646

**Table 5:** Comparative analysis of 24 h urine parameters across different sources.

	LabCorp, mmol/24 h	EAU Guidelines, mmol/24 h	Tietz Textbook, mmol/24 h
**Calcium**	male < 6.25/female < 5	<5.0	0.1 mmol·kg
**Oxalate**	0.2–0.45	<0.5	0.2–0.4
**Citrate**	male > 2.4/female > 2.9	male > 1.7/female > 1.9	male 0.6–4.8/female 1.3–6.0
**Uric Acid**	male < 4.8/female < 4.2	male < 5/female < 4	1.48–4.43
**Phosphorus**	20–40	<35	<32.3

## Data Availability

Datasets created during and/or analyzed during the current study are available from the relevant author upon reasonable request.
